# GNE Myopathy in Turkish Sisters with a Novel Homozygous Mutation

**DOI:** 10.1155/2016/8647645

**Published:** 2016-05-19

**Authors:** Gulden Diniz, Yaprak Secil, Serdar Ceylaner, Figen Tokucoglu, Sabiha Türe, Mehmet Celebisoy, Tülay Kurt İncesu, Galip Akhan

**Affiliations:** ^1^Department of Pathology, Neuromuscular Diseases' Centre, Tepecik Research and Training Hospital, Izmir, Turkey; ^2^Department of Neurology, Atatürk Research and Training Hospital, Izmir, Turkey; ^3^Department of Medical Genetics, Intergen Laboratory, Ankara, Turkey; ^4^Department of Neurology, Neuromuscular Diseases' Centre, Tepecik Research and Training Hospital, Izmir, Turkey; ^5^Department of Neurology, Katip Çelebi University, Izmir, Turkey

## Abstract

*Background*. Hereditary inclusion body myopathy is caused by biallelic defects in the GNE gene located on chromosome 9p13. It generally affects adults older than 20 years of age.* Methods and Results*. In this study, we present two Turkish sisters with progressive myopathy and describe a novel mutation in the GNE gene. Both sisters had slightly higher levels of creatine kinase (CK) and muscle weakness. The older sister presented at 38 years of age with an inability to climb steps, weakness, and a steppage gait. Her younger sister was 36 years old and had similar symptoms. The first symptoms of the disorder were seen when the sisters were 30 and 34 years old, respectively. The muscle biopsy showed primary myopathic features and presence of rimmed vacuoles. DNA analysis demonstrated the presence of previously unknown homozygous mutations [c.2152 G>A (p.A718T)] in the GNE genes.* Conclusion*. Based on our literature survey, we believe that ours is the first confirmed case of primary GNE myopathy with a novel missense mutation in Turkey. These patients illustrate that the muscle biopsy is still an important method for the differential diagnosis of vacuolar myopathies in that the detection of inclusions is required for the definitive diagnosis.

## 1. Introduction 

The GNE gene, located on 9p13, encodes the glucosamine (UDP-N-acetyl)-2-epimerase/N-acetylmannosamine kinase which is a key enzyme in sialic acid biosynthesis [1, 2]. The clinical hallmarks of GNE myopathy include late onset of symptoms, presence of distal involvement, and sparing of quadriceps muscle. In people with deficiency of GNE enzyme, the first step of sialic acid biosynthesis needed for the modification of proteins and fats cannot be achieved [2]. The biallelic mutations in the GNE gene cause the GNE myopathy (GNEM) which is a rare, progressive, autosomal recessive disease [3, 4]. GNE gene consists of 12 exons and its originally described transcript encodes 722 amino acids. In human, at least 6 different GNE transcripts have been described and it is still unknown which transcripts are crucial for causing GNEM [5]. The most important difference between these transcripts involves the first exon [5, 6].

GNEM is also known as Hereditary Inclusion Body Myopathy (HIBM), Distal Myopathy with Rimmed Vacuoles (DMRV), or Nonaka Myopathy. Muscle weakness typically starts around 20 to 30 years of age in patients with GNEM and the disease can lead to very severe disability over the following 10 to 20 years [4, 5]. There are no approved therapies for GNEM. But several therapy methods are under investigation such as the use of precursors of sialic acids or early gene therapy trials. Therefore the timely and accurate diagnosis is critical in patients with GNEM [6].

It is impossible to establish a diagnosis in patients with GNEM on clinical grounds alone. Therefore, muscle biopsy and molecular genetic analysis are mandatory for the correct diagnosis [3, 7]. Hitherto, over 150 GNE gene variants were reportedly associated with GNEM. Our patients' genotypes demonstrated a previously unknown missense mutation of GNE gene and this finding adds to the growing spectrum of mutations in the GNE gene.

## 2. Case Reports

### 2.1. Case  1

**Figure 1:**
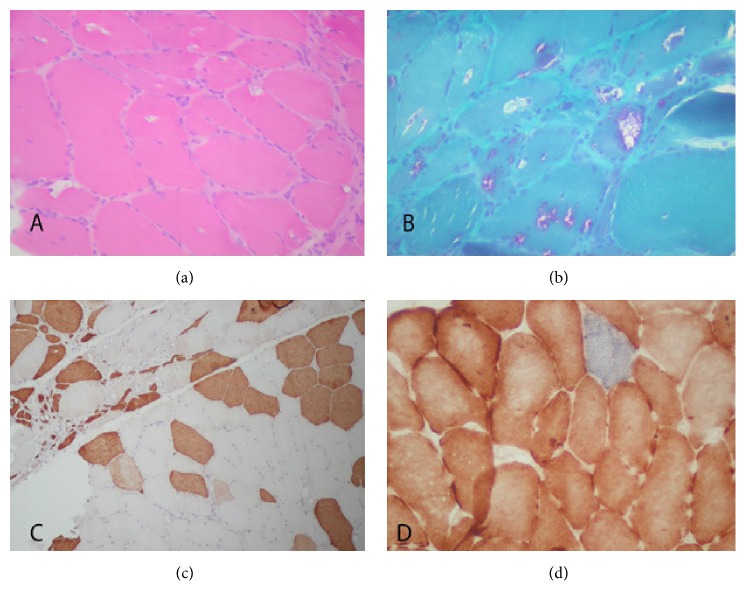
(a) Note the marked variation in fiber size and shape (HE ×200). (b) There are several rimmed vacuoles in the muscle biopsy specimen of Case  1 (Modified Trichrome ×200). (c) Note the plenty of immature fibers with neonatal myosin (DAB ×100). (d) Combined enzyme staining represents normal mitochondrial function (COX+SDH ×400).

A 38-year-old female presented with difficulty in climbing stairs, episodes of falling, and shortness of breath. She was 30 years old at the onset of her first symptoms of steppage walking. Neurological evaluation revealed proximal 4/5 and distal 4/5 muscle power in bilateral upper extremities and proximal 3/5 and distal 3/5 muscle power in bilateral lower extremities, while the strength of quadriceps femoris muscle was relatively spared. Deep tendon reflexes were hypoactive in all extremities. Full blood count, biochemical and thyroid function test results, sedimentation rate, C-reactive protein (CRP) levels, and vitamin B12 and D values were within normal limits. The CK value was 230 U/L (29–168 U/L). Results of electromyography (EMG) were consistent with myopathy. The vital capacity was low as revealed with respiratory function tests. Electrocardiography (ECG) and echocardiography findings were within normal ranges. The histopathological examination of muscle biopsy specimen taken from gastrocnemius muscle revealed myopathic features with several rimmed vacuoles (Figure 1). The family history of Case  1 revealed that her sibling had also similar problems.

### 2.2. Case  2

Case  2 was 36 years old and had similar symptoms. She was 34 years old at the onset of her first symptoms of foot drop. She presented two years after onset of the disease with difficulty in going upstairs, sitting, and standing with frequent episodes of falling. Neurological examination revealed proximal and distal 4/5 muscle strength in bilateral upper and lower extremities, while the strength of quadriceps femoris muscle was spared. Deep tendon reflexes were hypoactive in all extremities. Blood tests showed high CK levels (480 U/L), while other results were normal. EMG results were consistent with myopathy. Respiratory function test and blood gas evaluation results were normal. The ECG and echocardiography results were within normal limits on cardiology evaluation. First biopsy specimen obtained from gastrocnemius muscle revealed an end-stage muscle disease. In addition, the presence of group atrophy and angular fibers created the suspicion of neurogenic myopathy. The second biopsy from deltoid muscle had been also diagnosed as hereditary inclusion body myopathy.

**Figure 2:**
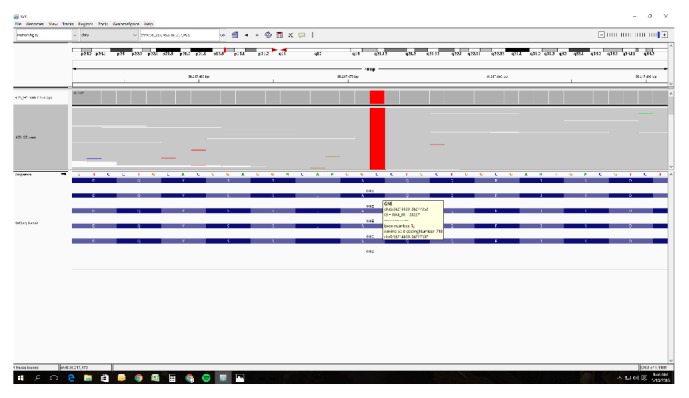
The homozygous GNE gene mutation in both sisters.

Five years after histopathological diagnosis, genetic tests were performed in both sisters and their families. DNA was extracted from peripheral lymphocytes. All exons and splice regions of the GNE gene (NM_001128227) were sequenced. We identified a new homozygous mutation on kinase domain of GNE gene in both sisters (Figure 2). This mutation (c.2152G>A/p.A718T) was of G-to-A transition at nucleotide position (c.2152G>A), which changes an amino acid at codon 718 from alanine (A) to threonine (T). Similar heterozygous mutations were found in their father and mother and in two daughters of the older sister, but GNE genes were normal in the other family members.

## 3. Discussion

Distal myopathies can be defined as a group of heterogeneous disorders classified into one broad category due to the presentation of weakness involving the distal skeletal muscles [1, 8]. GNEM is a late-adult onset autosomal recessive myopathy that is clinically characterized by progressive distal leg atrophy and weakness, especially involving lower limbs but sparing quadriceps group. Therefore it is also named as Distal Myopathy with Rimmed Vacuoles. The first symptom of GNEM is often foot drop, which is characterized by difficulty in lifting the front part of the foot, and dragging of the affected foot (feet) on the ground when walking. This situation was known as steppage walking. As additional muscles become affected by GNEM, difficulties in climbing stairs or getting up from a sitting position and weakness of the hands and shoulder muscles are observed. Patients with GNEM generally become lifetime wheelchair users within an average of 12 years after disease onset [1–3]. Interestingly, in our cases, atrophy and weakness not only involved muscles of the lower extremities but also affected the proximal muscles on both limbs which manifested 5 years after disease onset. Therefore clinically muscular dystrophies of the limb girdle should be suspected in the differential diagnosis.

Biopsies of the affected muscles show dystrophic changes of variable severity, including marked variation in fiber size, occasional fiber necrosis and regeneration, increased number of fibers with internal nuclei, and fiber splitting. Rimmed vacuoles or inclusions are present in the majority of patients, especially in the early stages of the disease. In late stages, muscle fibers are subsequently replaced by fat and fibrous tissue and the vacuoles are no longer discernible. In our second case, the first biopsy was from gastrocnemius muscle which demonstrated an end-stage muscle disease. Therefore the diagnosis could not be made based on the histopathological examination of the first biopsy specimen of the younger sister. Second biopsy was sampled from deltoid muscle which demonstrated many cellular inclusions. As there are only a few single case reports or relatively small case series regarding GNE myopathy, it is difficult to determine genotype-phenotype correlation. The largest ethnic cluster of GNEM was detected in the community of Jews migrating from and neighboring Middle Eastern countries [5]. An interesting founder mutation was also reported in a gypsy cohort. However, recently, Cho et al. [9] reported the mutation profile and clinical findings of the GNEM in 212 Japanese patients, which is the largest single-ethnic cohort. They identified 63 different mutations including 25 novel mutations. Fifty of them were missense mutations. The most frequent mutation in this cohort was c.1714G>C (p.Val572Leu), which accounts for 48.3% of total alleles. Homozygosity for this mutation resulted in more severe phenotypes with earlier onset and faster progression of the disease. But compound heterozygotes c.1714G>C (p.Val572Leu) mutations caused the milder phenotype. Similarly, the second most common mutation, c.527A>T (p.Asp176Val), seemed to be a mild mutation as the onset of the disease was observed much later. Moreover, in a significant number of c.527A>T homozygotes, clinically manifest disease was not observed [9–11]. Hitherto there are no presented cases with GNEM in Turkey; and c.2152G>A/p.A718T homozygous mutation has been firstly detected and fully described in our case report. The older sister became wheelchair-bound patient during 10 years. When we compare our cases in terms of clinical progression with already reported cohorts, we can see that our cases had a more severe disease course because of quickly progressive course. So the effect of this novel mutation on the deterioration of their clinical manifestations cannot be denied.

All features of GNEM are still unknown. Therefore each reported case will help to match the genotype with phenotype of GNEM. In addition, identification of each specific GNE mutation will contribute to the clinical diagnosis and treatment of GNEM.
